# Metformin upregulates mitophagy in patients with T2DM: A randomized placebo‐controlled study

**DOI:** 10.1111/jcmm.14834

**Published:** 2020-01-23

**Authors:** Shipra Bhansali, Anil Bhansali, Pinaki Dutta, Rama Walia, Veena Dhawan

**Affiliations:** ^1^ Department of Experimental Medicine and Biotechnology Postgraduate Institute of Medical Education and Research (PGIMER) Chandigarh India; ^2^ Department of Endocrinology Postgraduate Institute of Medical Education and Research (PGIMER) Chandigarh India

**Keywords:** metformin, mitophagy, nucleotide‐binding oligomerization domain‐like receptor family pyrin domain‐containing 3 (NLRP3), randomized controlled trial, type 2 diabetes mellitus

## Abstract

Impaired mitochondrial autophagy (mitophagy) and NLRP3 inflammasome activation have been incriminated in the pathogenesis of T2DM. Metformin besides being an insulin sensitizer also induces autophagy; however, its effect on mitophagy and NLRP3 activation in patients with T2DM still remains elusive. Forty‐five drug‐naïve T2DM patients with HbA_1C_ 7%‐9% (53‐75 mmol/mol) were randomly assigned to receive either metformin, voglibose, or placebo for 3 months, and were also recommended for lifestyle intervention programme (n = 15 each). Mitochondrial oxidative stress (MOS) parameters, qPCR and immunoblotting of mitophagy‐related markers (PINK1, PARKIN, MFN2, NIX, LC3‐II, LAMP2), p‐AMPKα (T172), and NLRP3 proteins, as well as transmission electron microscopy (TEM) for assessing mitochondrial morphology were performed in the mononuclear cells of study patients. Both metformin and voglibose showed a similar efficacy towards the reduction in HbA_1c_ and MOS indices. However, multivariate ANCOVA divulged that mRNA and protein expression of mitophagy markers, NLRP3 and p‐AMPKα (T172), were significantly increased only with metformin therapy. Moreover, PINK1 expression displayed a significant positive association with HOMA‐β indices, and TEM studies further confirmed reduced distortions in mitochondrial morphology in the metformin group only. Our observations underscore that metformin upregulates mitophagy and subsequently ameliorates the altered mitochondrial morphology and function, independent of its glucose‐lowering effect. Further, restoration of normal mitochondrial phenotype may improve cellular function, including β‐cells, which may prevent further worsening of hyperglycaemia in patients with T2DM.

## 1 INTRODUCTION

Type 2 diabetes mellitus (T2DM) is a multifactorial disorder, characterized by an ominous octet with predominant components being insulin resistance and β‐cell dysfunction.^1^ Mounting evidence reveals that with advancing duration of disease, there is a progressive and inexorable loss of β‐cell function and/or mass, which have been attributed to gluco‐lipotoxicity, rising oxidative stress and accelerated β‐cell apoptosis.^2^ Recently, selective mitochondrial autophagy (mitophagy) has also been proposed as an essential pro‐survival phenomenon for the sustenance of β‐cell health and function as well as improvement in insulin sensitivity.^3^

Mitophagy is an important cell‐reparative process that regulates the mitochondrial quality control through autophagic degradation of the defunct and/or damaged mitochondria. Hence, mitophagy has been considered as a ‘rescue’ mechanism that disrupts the vicious cycle of increased oxidative stress and consequent mitochondrial damage; and thus, it ought to play a protective role in certain diseases like T2DM and neurodegenerative disorders.^4^, ^5^ The process of mitophagy is modulated by different proteins including PTEN‐induced putative kinase 1 (PINK1), PARKIN*,* NIP3‐like protein X (NIX) and mitofusin‐2 (MFN2)*,* as well as the autophagic receptors like microtubule‐associated protein light chain 3 (LC3) and lysosome‐associated membrane protein‐2 (LAMP2).^6^ Mitophagy is triggered in response to various mitochondrial stressors, and the initial event includes the recognition of superfluous mitochondria, followed by their recruitment to the double‐membrane autophagic vesicles, and subsequent engulfment and degradation of mitochondrial cargo by autolysosomes. Furthermore, emerging evidence reveals that mitochondrial dysfunction underlies insulin resistance and β‐cell dysfunction in T2DM.^7^ Increased production of mitochondrial reactive oxygen species (ROS) results in the activation of the JNK pathway, which promotes the phosphorylation of serine residues of insulin receptor substrate (IRS1) proteins, instead of tyrosine residues, thereby impairing insulin signalling cascade. As the optimal mitochondrial function is essential for ATP generation; therefore, altered ATP/ADP ratio in β‐cells, as a consequence of impaired mitochondrial function, has been shown to be associated with reduced insulin secretion.^8^ Moreover, it has been proposed that overproduction of mitochondrial ROS is a potential mechanism that decreases the first phase of glucose‐induced insulin secretion.^9^ Work from Twig and his colleagues also reported that mitophagy regulates the mitochondrial turnover in β‐cells, which is critical for maintaining the mitochondrial homeostasis, function and survival of β‐cells, and the deregulation of this process may result in the progression of T2DM.^10^ In this regard, our previous study suggested that attenuated mitophagy, accompanied with increased mitochondrial oxidative stress in T2DM patients, might contribute to the worsening of hyperglycaemia in these patients.^11^

Recently, it has been postulated that inflammasome activation is triggered by several danger signals including infection, metabolic dysfunction and mitochondrial oxidative stress.^12^ One of the most extensively studied and best‐characterized inflammasome is nucleotide‐binding oligomerization domain‐like receptor family pyrin domain‐containing 3 (NLRP3), which acts as a molecular platform for triggering the activation of caspase‐1 and potent pro‐inflammatory cytokine IL‐1β.^13^ Intriguingly, pyroptosis, another form of inflammation‐mediated programmed cell death, occurs as a consequence of caspase‐1 activation leading to the release of pro‐inflammatory cytokine IL‐1β.^14^ However, a recent study by Cheng et al (2018) reported that regulated  pyroptosis suppressed the degree of inflammation during the initial stages of apical periodontitis (AP), while extensive pyroptosis led to aggravated inflammation and induced cell death in acute AP.^15^ Thus, the extent of pyroptosis determines the magnitude of inflammation, which has long been associated with the degree of insulin resistance and progression to T2DM.

Numerous clinical studies have reported that metformin (1,1‐dimethylbiguanide) is a potent insulin sensitizer and has been recommended as a frontline drug in the management of T2DM.^16^ Besides anti‐hyperglycaemic action, metformin also exerts its pleiotropic beneficial effects via activation of the energy sensor AMP‐activated protein kinase (AMPK). Recently, it has also been discovered that AMPK positively regulates autophagy, and metformin has been shown to activate autophagy via AMPK.^17^, ^18^ In addition, prior evidence also demonstrated that AMPK is involved in the inflammasome activation and pyroptosis in LPS‐primed macrophages.^19^ However, the molecular mechanism of action of metformin in the regulation of mitophagy and NLRP3 still remains to be largely explored.

Accumulating evidence reveals that mononuclear cells may act as potential surrogate marker for insulin‐sensitive sites, as these cells also express insulin receptors, and are easily accessible, as compared to the other insulin‐target tissues such as skeletal muscle and adipocytes, which involve an invasive extraction procedure.^20^ In addition, these systemically circulating cells readily respond to ambient glucose levels and have been used previously in a couple of studies to demonstrate the alterations in autophagy/mitophagy, and mitochondrial phenotype and function in patients with T2DM, hence providing insights into the pathogenesis of T2DM.^11^, ^21^, ^22^ Further, documentation of the presence of metformin transporter, that is human organic cation transporter 1 (hOCT1; also known as SLC22A1) on mononuclear cells, uncovers new vistas in elucidating the novel mechanistic insights into metformin's action in these cells.^23^ Therefore, the present study aimed to assess the role of metformin in the modulation of mitophagy and NLRP3 in the mononuclear cells of drug‐naïve patients with newly diagnosed T2DM (NDT2DM).

## 2 MATERIAL AND METHODS

### 2.1 Study participants

Seventy eligible participants were screened at the Department of Endocrinology, PGIMER, Chandigarh. The target study population included 45 drug‐naïve newly diagnosed T2DM (NDT2DM) patients with an age range 30‐55 years, duration of diabetes < 6 months, BMI > 25 kg/m^2^ and HbA_1c_ levels 7%‐9% (53‐75 mmol/mol). The diagnosis of T2DM was established according to the American Diabetes Association (ADA) guidelines.^24^ Certain changes were incorporated in the inclusion criteria after study commencement. Initially, we intended to recruit the patients with HbA_1c_ range 7%‐8%, which was later modified to 7%‐9%, as the recruitment was taking longer than expected. Further, we also assessed the effect of metformin on NLRP3 expression, in addition to mitophagy markers, as there is a scarcity of data in this area of research. Exclusion criteria comprised of patients on any treatment with oral anti‐hyperglycaemic agents, pregnancy, systemic illness and subjects with T1DM.

### 2.2 Study design

A 3‐month prospective, randomized, single‐blind, placebo‐controlled interventional trial was conducted in T2DM patients, and the trial was registered with the Clinical Trials Registry—India (CTRI/2017/04/008422, http://www.ctri.nic.in). SB enrolled the study participants, and a research assistant randomly assigned the patients to receive either metformin (2 g/d in divided doses; 30 mins before the meals), or voglibose (0.3 mg thrice/d; immediately before the three main meals), or matching placebo (2 g/d; 30 minutes before the meals) in a 1:1:1 ratio using a random allocation software. Voglibose was used as an active comparator against metformin to eliminate the confounding effect of reduced glucotoxicity on mitophagy. The patients were also advised for lifestyle modifications and were assigned to the respective interventions by the consulting physicians, after the initial work‐up at endocrine outpatient clinic, who were visiting primarily for the management of T2DM. Method of treatment allocation to the patients was concealed through the sequentially numbered sealed envelopes (Figure S1).

### 2.3 End‐points

The primary end‐point of the study was to achieve a target HbA_1C_ < 7% (53 mmol/mol) and to assess the effect on expression profiling of mitophagy‐related markers and NLRP3 following metformin and voglibose therapy. The secondary outcome included the alterations in anthropometric measurements and indices of glucose‐insulin homeostasis.

### 2.4 Methods

After overnight fasting, the sampling procedure was performed twice; at baseline and post‐intervention, between 8:30 and 9:30 am, for carrying out biochemical investigations, expression profiling of mitophagy‐related markers and mitochondrial morphology analysis by transmission electron microscopy (TEM).

### 2.5 Anthropometry

The patients were evaluated for detailed anthropometric parameters including height, weight, body mass index (BMI) (in kg/m^2^ calculated by measuring weight and height using a weighing machine and stadiometer), waist circumference (WC) by a non‐stretchable measuring tape and body fat percentage using body composition analyser (Tanita), at the time of enrolment and at 3 months.

### 2.6 Study drugs

Metformin and placebo were prepared as identical tablets by USV, India Ltd., Mumbai, and Cosmos Pharmaceuticals, India, respectively. Voglibose was provided by Novartis, India.

### 2.7 Biochemical parameters

Fasting plasma glucose (FPG) and 2‐h plasma glucose (2 hPG) (after 75 g of anhydrous glucose load), liver and renal function tests, and lipid profile were measured by an auto‐analyser, which showed a precision of <10% coefficient of variation (CV) (Roche Diagnostics). HbA_1c_ testing was done by high‐performance liquid chromatography (HPLC)–based method using Bio‐Rad D10 system, which had a precision <1.8% CV (Bio‐Rad). Thyroid function tests (T_4_, TSH and TPO), fasting plasma insulin (FPI) and C‐peptide were determined by an immunochemiluminescence assay (ICMA) using a hormone auto‐analyser with the precision of <5% CV (Roche). Insulin sensitivity was estimated using homeostasis model assessment of insulin resistance (HOMA‐IR) = FPI (μIU/mL) * FPG (mg/dL)/ 405, while homeostasis model assessment of β‐cell function (HOMA‐β %) was calculated using the standard formula: 360 * FPI (μIU/mL)/ FPG (mg/dL) – 63.^25^

### 2.8 Peripheral blood mononuclear cells (PBMCs) isolation

Approximately 10 mL of peripheral venous blood was withdrawn from the study patients after overnight fasting. Briefly, PBMCs were isolated using Ficoll‐Hypaque density‐gradient centrifugation method (Sigma‐Aldrich).^26^ Cells were collected and counted, and viability was assessed by trypan blue exclusion staining.

### 2.9 Measurement of mitochondrial ROS (mtROS) content and mitochondrial membrane potential (ΔΨm)

Briefly, the cells were harvested and incubated with MitoSOX™ Red (Molecular Probes; Invitrogen) and JC‐1 stain (BD Biosciences), respectively, at 37°C in a humidified 5% CO_2_ incubator, and were washed twice with 1× PBS and assay buffer for monitoring the mitochondrial superoxide production and membrane potential as previously described.^11^ Fluorescence intensity of MitoSox Red and the percentage of cells with collapsed membrane potential were determined using a FACS Aria II flow cytometer (Becton‐Dickinson).

### 2.10 qRT‐PCR analysis

Total cellular RNA was extracted from the freshly isolated PBMCs using TRIzol‐Chloroform method (Invitrogen), and 2 μg cDNA was synthesized by reverse‐transcription PCR using a mastercycler gradient (Eppendorf). Transcriptional expression of mitophagy‐related genes including *PINK1, PARKIN, NIX, MFN2, LC3‐II* and *LAMP2* (primer sequences listed in Table S3) was performed on StepOnePlus real‐time PCR system (Applied Biosystems) using SYBR green chemistry as described previously.^11^ The precision of the real‐time instrument had a maximum cut‐off of 11% CV. 1 μL of template cDNA (equivalent to 100 ng of RNA) was added to a total 10 μL PCR mixture. All the reactions were run in duplicate alongside a no‐template control as a negative control. Relative quantification of gene expression was determined by the 2^−ΔΔCT^ method, and β‐actin was used as an endogenous control.^27^

### 2.11 Immunoblotting

Cell lysates were prepared, followed by SDS gel electrophoresis, and the separated proteins were transferred onto a PVDF membrane as per the protocol mentioned earlier.^11^ The membrane was blocked with 5% BSA for 1 hour and subsequently incubated overnight at 4°C with primary antibodies (Table S2). Thereafter, the membranes were washed and exposed with their corresponding HRP‐conjugated secondary antibodies for 1 hour. The membrane was rinsed thrice with TBST and treated with luminol reagent solution for 1 minute. The blots were then imaged using a ChemiDoc system (FluorChem M, Protein simple). The data were analysed using Image J (1.47 v) software to obtain the band intensity ratio of target proteins relative to β‐actin.

### 2.12 Transmission electron microscopy (TEM)

For ultra‐structural analysis of mitochondria, PBMCs were fixed with 3% glutaraldehyde, followed by re‐fixation in 1% OsO4, and were subsequently dehydrated with different grades of ethanol (70, 90 and 100%), prior to embedding in Epon resins. Ultrathin sections (50 nm) were obtained with a diamond knife, counterstained with uranyl acetate and were imaged on a JEM‐1400Plus electron microscope (JEOL Ltd.).^11^ Representative 30‐35 images of mitochondria from three patients per group were attained at baseline and post‐intervention. Evaluation of mitochondrial morphological characteristics including aspect ratio (major axis/minor axis), form factor (perimeter^2^/4π*Area), circularity (inverse of FF), interconnectivity (area/perimeter) and scoring for mitochondrial cristae appearance, was performed by two independent investigators in a blinded manner. Further, the average number of mitochondria per cell was calculated, and based on these findings, the percentage of damaged mitochondria in the study groups was determined.

### 2.13 Power and sample size calculation

Based on the large effect size (0.55) for ANCOVA with six covariates, a minimum sample size (n = 36) was calculated to achieve a power of ≥80% (G‐ power v3.1.9.2), with α = .05. Therefore, we planned to randomize 45 patients, 15 in each group, to ensure adequate study power even after drop‐outs.

### 2.14 Statistical analysis

We used parametric or non‐parametric test based on the distribution of the data. The normality of the distribution of variables was checked by measures of the Kolmogorov‐Smirnov test of normality. Data with normal distribution are represented as mean ± SD, and those with skewed distribution are expressed in median and interquartile range. Differences between pre‐ and post‐intervention values within a group were analysed by paired *t* test for parametric data or Wilcoxon's signed‐rank test for non‐parametric variables. Spearman and Pearson correlation coefficients were calculated to determine the association between two variables. Regression analysis was also performed to establish an association of mitophagy markers with HOMA‐β index. Analysis of co‐variance (ANCOVA) was employed to analyse the difference in the effect of 3 different interventions. In univariate ANCOVA, post‐intervention measurements were treated as the dependent variables, while the baseline parameters and the major confounding variables were added step‐wise in the model as covariates, after confirming that no multicollinearity existed between these covariates. Also, the intervention categories were taken as independent variables in the above model. Further, the effect size was calculated from partial eta‐squared, and based on Cohen's recommendation, eta‐squared values of 1%, 6% and 13.8% are suggestive of small, medium and large effect sizes, respectively.^28^ All the tests used a CI of 95% and values with *P* < .05 were implied statistically significant. All results obtained in the study were analysed using SPSS (version 22) and GraphPad Prism (version 6).

### 2.15 Study approval

The study was performed in accordance with the principles outlined in the Declaration of Helsinki, and the clinical trial protocol was approved by the Institutional Ethics Committee at PGIMER, Chandigarh, India. A written informed consent was obtained from the participants prior to their inclusion in the study.

## 3 RESULTS

### 3.1 Baseline characteristics of the study patients

The mean age and anthropometric measurements were comparable among all the study groups (Table 1). All patients were treatment‐naïve for anti‐diabetic medications, while one‐third of these patients received antihypertensive drugs. The mean duration of diabetes of the study participants was 3 months. Further, the glycemic variables including FPG and 2hPG levels were found to be significantly higher in patients in the placebo group as compared to the metformin‐ and voglibose‐treated group (*P* < .05). However, the mean HbA_1C_ levels were comparable among all the patients, suggesting a homogeneity in cumulative glycemic burden. Further, HOMA‐IR, HOMA‐β indices and lipid profile did not significantly differ among the study participants (Table 1).

**Table 1:** Baseline clinical and biochemical characteristics of the patients with NDT2DM

Parameters	METFORMIN (n = 15)	VOGLIBOSE (n = 15)	PLACEBO (n = 15)	*P*‐value
Age (years)	47.8 ± 7	45.5 ± 8.7	44.07 ± 8.4	.345
Gender (M:F)	7:8	6:9	8:7	‐
Ht. (cm)	155.0 ± 9.5	157.8 ± 9.2	156.2 ± 8.1	.708
Wt. (kg)	70.1 ± 11.1	74.4 ± 9.3	72.9 ± 8.3	.468
BMI (kg/m^2^)	29.3 ± 3.4	29.9 ± 3.4	29.9 ± 3.5	.824
WC (cm)	95.7 ± 5.3	99.3 ± 6.5	97.0 ± 8.6	.361
Body fat (%)	35.6 ± 10.6	37.5 ± 9.3	39.2 ± 8.9	.596
Systolic BP (mm Hg)	126.0 ± 12.9	127.3 ± 10.9	122.0 ± 10.8	.434
Diastolic BP (mm Hg)	82.0 ± 8.6	88.0 ± 7.4	80.4 ± 7.1	.028*
FPG (mg/dL)	138.4 ± 17.2	140.9 ± 21.4	160.2 ± 22.1	.010**
2hPG (mg/dL)	255.4 ± 57.5	241.4 ± 58.9	299.5 ± 58.9	.025*
HbA_1C_ (%)	7.8 ± 0.4	7.6 ± 0.5	7.7 ± 0.4	.664
FPI (μIU/mL)	13.7 ± 5.3	15.9 ± 6.0	13.7 ± 5.1	.438
HOMA‐IR	4.8 ± 2.2	5.5 ± 2.1	5.6 ± 2.8	.588
HOMA‐β %	67.2 ± 27.7	69.9 ± 34.1	50.3 ± 14.0	.084
S. CHOL (mg/dL)	188.5 ± 35.6	205.0 ± 40.7	204.0 ± 25.5	.355
S. LDL‐C (mg/dL)	125.1 ± 33.8	137.7 ± 31.0	133.7 ± 27.5	.549
S. TG (mg/dL)	141.0 ± 41.9	159.3 ± 49.0	160.1 ± 66.6	.559
S. HDL‐C (mg/dL)	41.9 ± 6.4	41.2 ± 6.6	46.6 ± 13.5	.277

Note

Data are expressed as Mean ± SD.

* *P* < .05;

** *P* < .01.

### 3.2 Effect of metformin and voglibose on clinical and biochemical characteristics of the study patients

On intra‐group comparison, we observed that both metformin and voglibose treatment led to a significant decrease in the anthropometric parameters including weight, BMI, waist circumference (WC) and body fat percentage (%) (*P* < .05). Also, a significant reduction in the glycemic variables (FPG, 2hPG, HbA_1c_ and HOMA‐IR levels) was observed in both the groups (*P* < .05). However, HOMA‐β indices were insignificantly higher after 3 months of both metformin and voglibose therapy. Following placebo therapy, a significant decrease in weight, BMI, WC, 2hPG and HOMA‐IR levels were observed at 3 months relative to their baseline values (*P* < .05) (Table 2).

**Table 2:** Clinical and biochemical characteristics of the patients with NDT2DM at baseline and 3 mo

Parameters	Metformin			Voglibose			Placebo		
	Baseline (n = 14)	3 mo (n = 14)	*P*‐value	Baseline (n = 15)	3 mo (n = 15)	*P*‐value	Baseline (n = 13)	3 mo (n = 13)	*P*‐value
Wt. (kg)	70.2 ± 11.5	65.6 ± 11.1	.0001***	74.4 ± 9.3	70.3 ± 8.5	.0001***	72.8 ± 8.8	70.2 ± 9.8	.009**
BMI (kg/m^2^)	29.4 ± 3.5	27.3 ± 3.9	.0001***	29.9 ± 3.4	28.4 ± 3.3	.0001***	29.7 ± 3.6	28.8 ± 3.9	.016*
WC (cm)	94.8 ± 4.3	90.9 ± 7.0	.004**	99.3 ± 6.5	93.9 ± 6.4	.0001***	96.1 ± 8.5	93.4 ± 9.1	.013*
Body Fat (%)	35.2 ± 10.9	33.0 ± 11.3	.012*	37.6 ± 9.3	35.2 ± 9.9	.0001***	38.9 ± 9.5	38.1 ± 8.7	.568
FPG (mg/dL)	137.6 ± 17.6	114.1 ± 15.5	.007**	140.9 ± 21.4	116.6 ± 14.9	.0001***	159.1 ± 22.6	151 ± 46.7	.357
2hPG (mg/dL)	250.1 ± 55.6	148.1 ± 41.1	.0001***	241.5 ± 58.9	147.3 ± 35.5	.0001***	298.2 ± 60.9	239.1 ± 70.8	.012*
HbA_1C_ (%)	7.7 ± 0.4	6.4 ± 0.4	.0001***	7.6 ± 0.5	6.4 ± 0.5	.0001***	7.7 ± 0.4	7.9 ± 1.2	.434
HOMA‐IR	4.5 ± 2.2	2.7 ± 1.3	.030*	5.5 ± 2.1	3.5 ± 1.2	.0001***	5.8 ± 2.7	4.6 ± 2.9	.045*
HOMA‐β (%)	67.2 ± 28.8	74.7 ± 37.1	.408	69.9 ± 34.1	74.2 ± 21.1	.609	52.1 ± 12.6	53.9 ± 26	.793

Note

Data are expressed as Mean ± SD.

* *P* < .05;

** *P* < .01;

*** *P* < .001 vs corresponding baseline values.

### 3.3 Comparison of ∆ change (pre‐ and post‐intervention) in clinical and biochemical parameters of NDT2DM patients

On inter‐group comparison, we observed that both metformin and voglibose treatment led to a significant reduction in HbA_1C_ levels, as compared to the placebo group (*P* < .05). Furthermore, 92% and 80% of the NDT2DM patients receiving either metformin or voglibose therapy achieved HbA_1C_ target levels <7% as compared with up to 30% of the placebo‐treated group. However, no significant changes in anthropometric measurements, glycemic profile, HOMA‐IR and HOMA‐β indices were observed in the metformin‐ and voglibose‐treated group, when compared with the placebo group (Table 3).

**Table 3:** Pre‐ and post‐comparison (Δ change) in the clinical and biochemical characteristics of study patients

Variables	Metformin group (n = 14)	Voglibose group (n = 15)	Placebo group (n = 13)	Met vs Vogli *P*‐value	Met vs Pbo, *P*‐value	Vogli vs Pbo, *P*‐value
∆ Wt. (kg)	−4.6 (−6.2 to −2.5)	−4.1 (−5.8 to −2.5)	−3.4 (−3.9 to 0)	1.000	.277	.863
∆ BMI (kg/m^2^)	−1.6 (−2.7 to −1.2)	−1.6 (−1.9 to −0.9)	−0.9 (−1.5 to 0)	.528	.643	.709
∆ WC (cm)	−3.3 (−6.5 to −1.6)	−5 (−7.7 to −3.6)	−1 (−5.7 to −0.2)	.525	1.000	.082
∆ Body fat (%)	−2.2 (−3.3 to −0.5)	−2.4 (−3.1 to −1.8)	−1.7 (−3.4 to 1.3)	1.000	1.000	.747
∆ FPG (mg/dL)	−31.7 (−45.1 to −2.5)	−24 (−36.3 to −11.7)	−14.5 (−21.3 to 0.5)	1.000	.351	.289
∆ 2hPG (mg/dL)	−102.7 (−138.1 to −56.7)	−95.5 (−130.7 to −53)	−77.3 (−109.3 to −1.5)	1.000	.292	.496
∆ HbA_1C_ (%)	−1.4 (−1.6 to −1)	−1.2 (−1.4 to −0.9)	0 (−0.5 to 0.9)	1.000	.0001***	.0001***
∆ HOMA‐IR	−1.6 (−3.3 to −0.5)	−2 (−3 to −0.9)	−1.8 (−2.3 to 0.3)	1.000	1.000	1.000
∆ HOMA‐β (%)	+5.6 (−11.3 to 26.9)	+15.3 (−13.8 to 21.4)	−4.6 (−17.5 to 7.5)	1.000	1.000	1.000

Note

Data are expressed as median and interquartile range.

*** *P* < .001.

### 3.4 Effect of metformin and voglibose on mitochondrial ROS (mtROS) content mitochondrial membrane potential (MMP)

**Figure 1:**
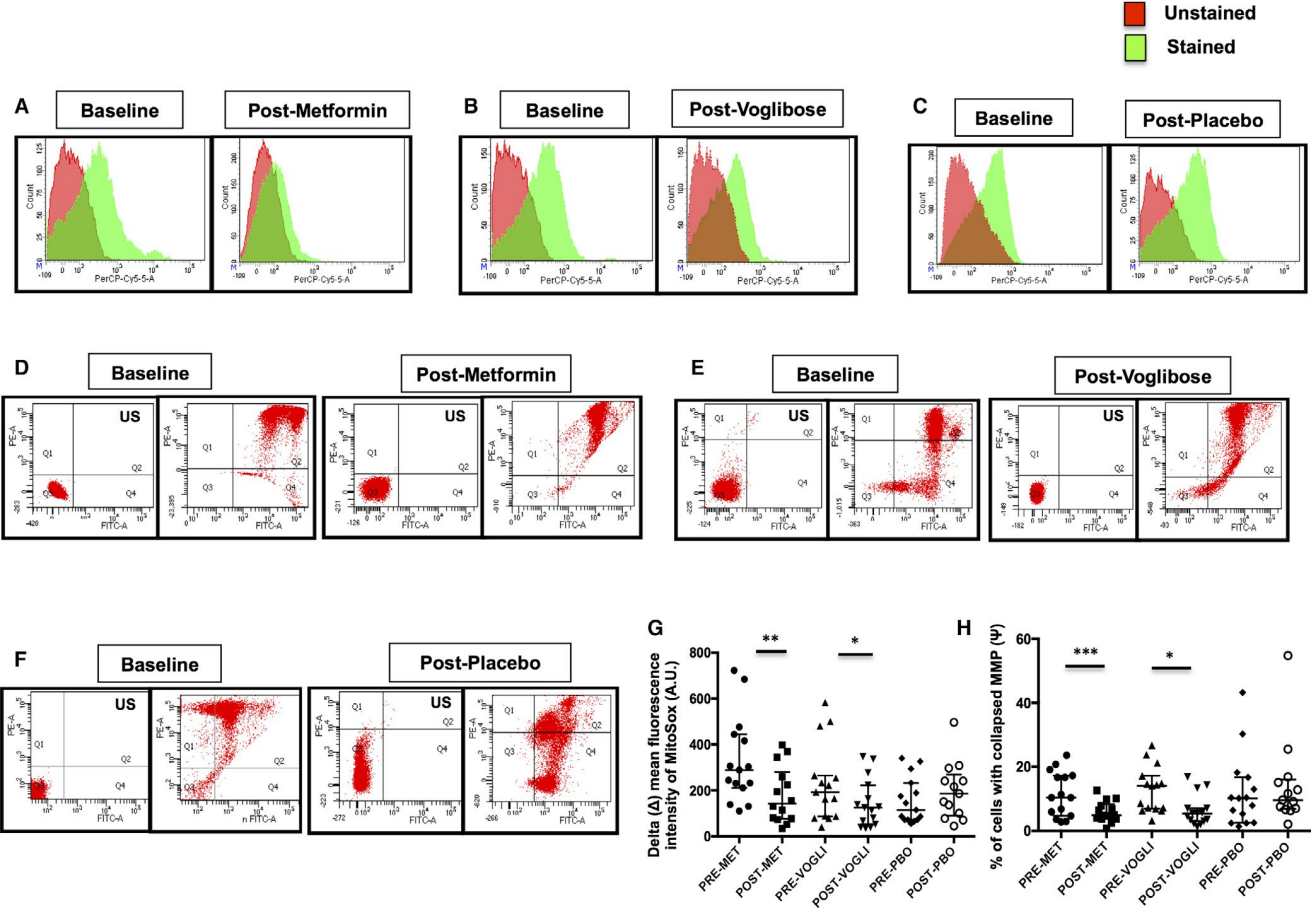
Representative flow cytometry histogram overlays of MitoSox Red fluorescence intensity in NDT2DM patients at baseline and at 3‐mo follow‐up, US—unstained (autofluorescence), (A) metformin‐treated group, (B) voglibose‐treated group and (C) placebo‐treated group. Representative dot plots illustrating the mitochondrial membrane potential (MMP) by JC‐1 using flow cytometry, US—unstained (autofluorescence), (D) metformin‐treated group, (E) voglibose‐treated group and (F) placebo‐treated group. (G & H) Scatter plot represents delta (Δ) mean fluorescence intensity of MitoSox Red and the percentage of cells with collapsed MMP in the respective groups (n = 13‐15 each). Values are expressed in median and interquartile range; *P < .05, **P < .01, ***P < .001 vs baseline, Wilcoxon signed‐rank test, as indicated

Intra‐group comparison revealed that both mtROS production and % of cells with collapsed MMP were significantly decreased following metformin and voglibose treatment relative to their baseline levels (*P* < .05), while the placebo‐treated subjects presented an insignificant rise in the mtROS content and collapsed MMP (Figure 1). However, on the inter‐group comparison, the univariate baseline model revealed no significant difference in the mtROS content and MMP after 3 months of the respective therapies (Table S1).

### 3.5 Changes in the expression levels of mitophagy‐related markers following metformin and voglibose therapy

#### 3.5.1 Intra‐group comparison

**Figure 2:**
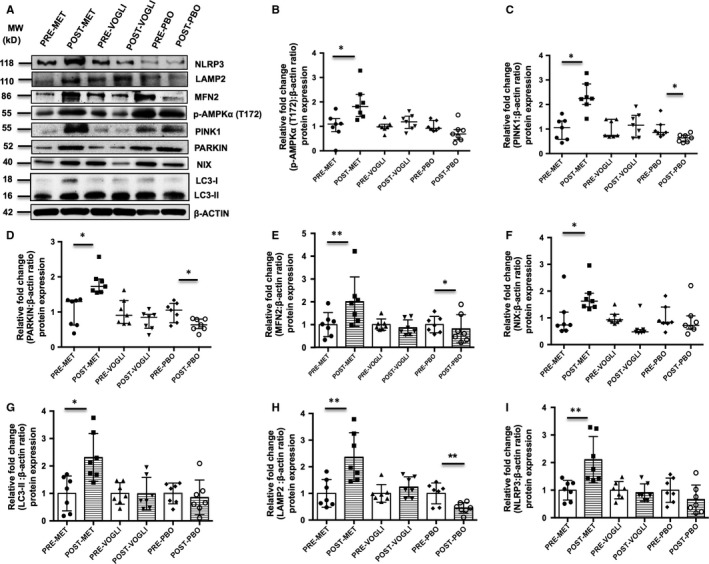
A, Representative Western blots of NLRP3, p‐AMPKα (T172) and mitophagy‐related proteins; β‐actin was used as a loading control. Quantification of protein expression of (B) p‐AMPKα (T172), (C) PINK1, (D) PARKIN, (E) MFN2, (F) NIX, (G) LC3‐II, (H) LAMP2 and (I) NLRP3, in NDT2DM patients at baseline and following treatment with metformin, voglibose and placebo, respectively. All the values of the baseline groups were set to 1, and the expression levels for the treatment groups were derived relative to their corresponding controls (baseline), and the values have been expressed in fold change. Scatter plot displays values in median and interquartile range, while scatter plot with bar diagram represents the values in Mean ± SD (n = 7 each). *P < .05; **P < .01, *P*‐values were calculated using Paired *t* test or Wilcoxon signed‐rank test

We observed that metformin significantly augmented the relative mRNA expression of the mitophagy genes, that is *MFN2*, *NIX, PARKIN, PINK1, LC3‐II* and *LAMP2* by ~2‐ to 3.5‐fold (*P* < .05). However, voglibose‐ and placebo‐treated study subjects exhibited no significant effect on the mRNA expression of these aforementioned genes (Figure S2). Consistent with the mRNA expression pattern, a similar protein expression profile of these mitophagy markers was observed in the metformin‐treated group as compared to the voglibose and placebo‐treated groups (*P* < .05). Interestingly, patients receiving metformin also demonstrated a significant increase in p‐AMPKα (T172) and NLRP3 protein levels, in contrast to the voglibose and placebo groups (*P* < .05) (Figure 2).

#### 3.5.2 Inter‐group comparison

Uni‐ and multivariate analysis among the three groups for various outcome measures have been depicted in Tables 4 and 5. On univariate ANCOVA analysis, transcriptional expression of mitophagy‐related markers (*MFN2*, *PARKIN, PINK1, LC3‐II* and *LAMP2)* was found to be significantly augmented with metformin, except for *NIX* mRNA levels, and this observation persisted even after adjusting for age, BMI, HbA_1C,_ HOMA‐IR and HOMA‐β indices (*P* < .05 each) in the final model (Table 4). Along the similar lines, protein expression trend for mitophagy indicators, p‐AMPKα (T172) and NLRP3, showed a significant increase with metformin treatment (*P* < .05 for each), even after adjusting for all the major confounders, using both univariate and multivariate analysis (Table 5).

**Table 4:** Comparative efficacy of metformin vs voglibose and placebo on mRNA expression of mitophagy‐related markers

	Baseline Model (n = 42)					Controlling for age		Controlling for age, BMI		Controlling for age, BMI, HbA_1c_		Controlling for age, BMI, HbA_1c_, HOMA‐IR		Controlling for age, BMI, HbA_1c_, HOMA‐IR, HOMA‐β				
	*P*‐Value	Effect size (%)^a^	Adjusted 3‐mo mean^b^ (95% CI)			*P*‐value	Effect size (%)^a^	*P*‐value	Effect size (%)^a^	*P*‐value	Effect size (%)^a^	*P*‐value	Effect size (%)^a^	*P*‐value	Effect size (%)^a^	Adjusted 3‐mo mean^b^ (95% CI)		
																Met	Vogli	Placebo
			Met	Vogli	Placebo													
PINK1	.002	28.5	2.12 (1.63, 2.60)	1.06 (0.59, 1.53)	0.89 (0.39, 1.40)	.002	28.8	.002	28.9	.002	29.1	.006	28.1	.001	38.9	2.32 (1.76, 2.88)	1.22 (0.67, 1.77)	0.67 (0.09, 1.25)
PARKIN	.004	24.8	2.26 (1.62, 2.91)	1.05 (0.42, 1.67)	0.75 (0.08, 1.41)	.002	28.0	.004	26.9	.003	28.1	.001	34.3	.004	33.8	2.57 (1.83, 3.30)	1.13 (0.41,1.84)	0.71 (−0.06,1.48)
NIX	.161	9.2	1.60 (1.11, 2.09)	1.23 (0.75, 1.70)	0.92 (0.41, 1.42)	.145	9.9	.187	8.9	.186	9.2	.421	5.4	.304	8.4	1.58 (1.00, 2.15)	1.23 (0.68, 1.79)	0.91 (0.32,1.50)
LC3‐II	<.001	33.4	2.24 (1.71, 2.77)	0.75 (0.23, 1.26)	0.96 (0.41, 1.52)	.001	32.4	.001	33.1	.001	32.9	<.001	42.7	.001	38.8	1.95 (1.51, 2.39)	0.78 (0.34, 1.21)	0.95 (0.48, 1.41)
LAMP2	<.001	45.4	3.62 (2.87, 4.36)	1.26 (0.55, 1.98)	0.93 (0.16, 1.70)	<.001	44.7	<.001	44.6	<.001	44.5	<.001	44.8	<.001	45.5	3.72 (2.82, 4.62)	1.37 (0.50, 2.25)	0.87 (−0.07, 1.80)
MFN2	<.001	45.9	2.61 (2.09,3.12)	0.99 (0.49,1.48)	0.71 (0.18,1.25)	<.001	49.3	<.001	48.6	<.001	48.6	<.001	52.7	<.001	52.2	2.87 (2.26, 3.49)	0.95 (0.35, 1.56)	0.71 (0.07, 1.36)

a Variance explained by the use of metformin; calculated from partial eta‐squared. Squared values of 1%, 6% and 13.8% indicate small, medium and large effect sizes, respectively.

b Adjusted 3‐mo mean for outcome variables calculated controlling for effects of confounder(s).

**Table 5:** Comparative efficacy of metformin vs voglibose and placebo on protein expression of mitophagy‐related markers, p‐AMPKα (T172) and NLRP3

	Baseline model (n = 21)					Controlling for age		Controlling for age, BMI		Controlling for age, BMI, HbA_1c_		Controlling for age, BMI, HbA_1c_, HOMA‐IR		Controlling for age, BMI, HbA_1c_, HOMA‐IR, HOMA‐β				
	*P*‐value	Effect size (%)^a^	Adjusted 3‐mo mean^b^ (95% CI)			*P*‐value	Effect size (%)^a^	*P*‐value	Effect size (%)^a^	*P*‐value	Effect size (%)^a^	*P*‐value	Effect size (%)^a^	*P*‐value	Effect size (%)^a^	Adjusted 3‐mo mean^b^ (95% CI)		
																Met	Vogli	Placebo
			Met	Vogli	Placebo													
PINK1	<.001	75.6	2.33 (1.97, 2.69)	1.18 (0.82, 1.54)	0.63 (0.28, 0.99)	<.001	75.8	<.001	75.9	<.001	76.2	.001	69.2	.004	63.9	2.05 (1.59, 2.51)	1.23 (0.84, 1.61)	0.69 (0.28, 1.10)
PARKIN	<.001	82.8	1.83 (1.62, 2.05)	0.75 (0.53, 0.97)	0.64 (0.42, 0.85)	<.001	82.8	<.001	83.4	<.001	84.9	<.001	90.8	<.001	90.1	2.03 (1.79, 2.28)	0.66 (0.46, 0.86)	0.60 (0.38, 0.82)
NIX	.002	51.3	1.77 (1.35, 2.19)	0.63 (0.21, 1.04)	0.92 (0.51, 1.34)	.003	52.2	.003	53.8	.004	54.1	.053	38.7	.053	41.4	1.77 (1.12, 2.42)	0.66 (0.11, 1.21)	0.94 (0.34, 1.54)
LC3‐II	.002	50.7	2.31 (1.74, 2.89)	0.99 (0.42, 1.57)	0.86 (0.28, 1.43)	.003	50.7	.005	50.6	.007	50.6	.093	33.0	.162	28.2	2.08 (1.22, 2.93)	1.01 (0.34, 1.69)	1.12 (0.38, 1.87)
LAMP2	<.001	70.4	2.36 (1.91, 2.81)	1.24 (0.79, 1.69)	0.46 (0.01, 0.90)	<.001	71.7	<.001	72.7	<.001	73.7	<.001	75.1	.001	71.9	2.49 (1.91, 3.07)	1.12 (0.64, 1.60)	0.41 (−0.12,0.93)
MFN2	<.001	74.5	2.01 (1.72, 2.29)	0.87 (0.58, 1.15)	0.81 (0.53, 1.10)	<.001	75.1	<.001	75.3	<.001	76.8	.001	70.0	.002	69.0	2.03 (1.61, 2.45)	0.93 (0.58, 1.27)	0.72 (0.34, 1.10)
NLRP3	<.001	60.4	2.11 (1.65, 2.56)	0.91 (0.46, 1.36)	0.66 (0.21, 1.11)	<.001	65.3	<.001	65.1	<.001	69.4	.001	69.2	.002	66.6	2.21 (1.68, 2.74)	0.77 (0.33, 1.21)	0.81 (0.33, 1.29)
p‐AMPKα (T172)	.001	58.5	1.94 (1.57, 2.31)	1.17 (0.80, 1.54)	0.75 (0.38, 1.12)	.001	58.7	.001	58.6	.001	62.6	.005	58.3	.007	59.7	2.05 (1.58, 2.51)	1.08 (0.70, 1.46)	0.83 (0.41, 1.25)

a Variance explained by the use of metformin; calculated from partial eta‐squared. Squared values of 1%, 6% and 13.8% indicate small, medium and large effect sizes, respectively.

b Adjusted 3‐mo mean for outcome variables calculated controlling for effects of confounder(s).

### 3.6 Correlation and multiple linear regression analysis of various parameters following different treatments

Intriguingly, a significant and positive correlation was observed between the increased NLRP3 and p‐AMPKα (T172) protein levels (*r* = .886; *P* = .008) in the metformin‐treated group. In addition, HOMA‐β indices showed a positive and significant correlation with NLRP3 expression in the placebo group (*r* = .821; *P* = .023). Moreover, univariate linear regression analysis revealed that both PINKI and BMI were significantly and independently associated with the HOMA‐β indices (*P* < .05) in the metformin group. Furthermore, even after adjustment for BMI, hierarchical linear regression analysis showed that PINK1 levels still maintained the statistical significance and contributed to 31% of the variance in the HOMA‐β indices in the metformin‐treated group (*P* = .001).

### 3.7 Alterations in the mitochondrial morphology following metformin and voglibose therapy

**Figure 3:**
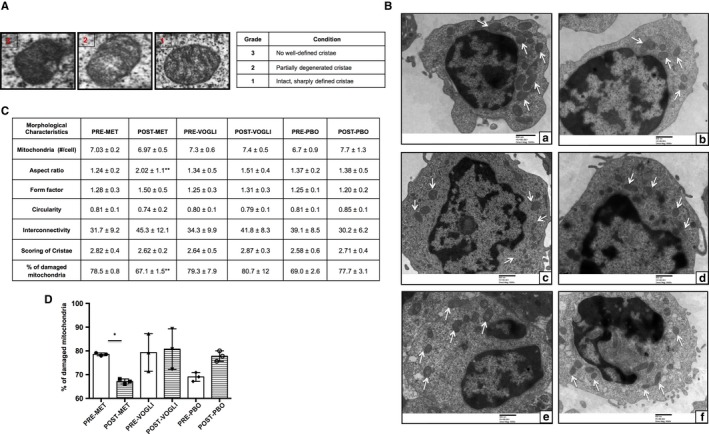
A, Images representing the scoring system ranging from 1 (best) to 3 (worst), depending upon the cristae appearance. B, Representative electron micrographs showing mitochondria (white arrows) observed in (a & b) pre– and post–metformin‐treated patients, (c & d) pre– and post–voglibose‐treated patients and (e & f) pre– and post–placebo‐treated patients. Scale bars are indicated. Original magnification: A to E, 5000×, (C). Table depicting the mitochondrial morphological characteristics and (D) scatter plot with bar diagram displaying the percentage of damaged mitochondria in patients with NDT2DM at baseline and post‐follow‐up. Values are expressed in Mean ± SD (n = 3 each group). *P < .05 vs baseline

Electron micrographs revealed that the metformin‐treated group displayed significantly decreased accumulation of damaged mitochondria (*P* < .05), while we observed no difference in the percentage of distorted mitochondria in the voglibose group. Further, in placebo‐treated study patients, an increase in the % of damaged mitochondria was observed at 3 months, though the difference did not attain any statistical significance. Also, analysis of major mitochondrial morphology descriptors indicated that form factor, circularity and interconnectivity differed post‐treatment in all the groups, though insignificantly, except for the aspect ratio, which was observed to be significantly increased in the metformin‐treated group (*P* < .05) (Figure 3).

## 4 DISCUSSION

The present interventional study reveals that metformin exerts its beneficial effect on mitochondrial health via promoting mitophagy‐mediated clearance of damaged mitochondria in patients with T2DM. This observation signifes that upregulation of mitophagy preserves mitochondrial morphology and function, ameliorates oxidative stress, which may subsequently alleviate worsening of hyperglycemia and its related complications in patients with newly diagnosed T2DM receiving metformin therapy. In addition, our findings uncover novel insights into the action of metformin on mitochondrial quality control mechanism, besides its antihyperglycaemic effect.

Evidences in the literature report that uncontrolled hyperglycaemia for a long‐term in individuals with an advanced duration of T2DM (>10 years) causes irreversible damage to the cell repair processes like mitophagy, subsequently resulting in the accumulation of superfluous mitochondria. These events are accompanied with a concomitant increase in mitochondrial oxidative stress, which eventually results in the manifestation and progression of micro and macro‐angiopathic complications in these patients.^29^ However, patients with newly diagnosed T2DM (NDT2DM) present with hyperglycaemia for a shorter‐duration, and early and intensive management of T2DM in these patients helps to restore euglycemia rapidly, which may exert a protective effect on mitochondrial phenotype, thereby reducing the long‐term risk of developing diabetes‐related complications. Furthermore, we aimed to assess the exclusive effect of metformin on mitophagy, as metformin is a first‐line therapy recommended for the management of NDT2DM. On the contrary, patients with long‐standing diabetes require polytherapy (oral anti‐diabetic drugs (OADs) and/or insulin), which may not provide an opportunity to explore the sole effect of metformin on mitophagy in these patients. Therefore, we preferred the inclusion of treatment‐naïve patients of NDT2DM with a duration of diabetes < 6 months for our study.

It is well known that impaired mitophagy leads to the accelerated progression of mitochondrial dysfunction, which has been implicated in the aggravation of insulin resistance and deterioration of β‐cell function in patients with T2DM.^30^ Metformin is recommended as the first‐line drug in the management of T2DM, and its effect on autophagy is well explored; however, there is a dearth of literature regarding its impact on mitophagy.^18^, ^31^ Furthermore, voglibose, an alpha‐glucosidase inhibitor, which neither has a direct effect on insulin sensitivity nor acts as a β‐cell secretagogue, was employed as an active comparator against metformin to eliminate the confounding influence of reduced glucotoxicity on mitochondrial health.^32^

Our data demonstrated that both metformin and voglibose exhibited an equal glucose‐lowering effect as corroborated by a significant reduction in FPG, 2hPG, and HbA_1C_ levels, indicating that the two drugs appeared to have similar efficacy . Further, a significant weight loss was observed in all the study participants, depicting that lifestyle modification programme played a prominent role in weight reduction in these patients. The rationale behind this observation is supported by the prior reports, which have demonstrated that both metformin and voglibose are either weight‐neutral or induce modest weight loss in T2DM patients.^33^, ^34^, ^35^ Additionally, metformin use is invariably associated with improvement in HOMA‐IR levels, as was also observed in our study. However, the similar findings were noted in the voglibose and the placebo group, which can be attributed to the comparable magnitude of weight reduction in these participants, hence negating the insulin‐sensitizing effect of metformin. Intriguingly, the HOMA‐β indices were found to be modestly higher in the metformin‐treated group, which could be ascribed to the reduction in glucotoxicity, and glucagon‐like peptide 1 (GLP‐1)‐mediated improvement in β‐cell function, as metformin also stimulates GLP‐1 secretion.^36^ Further, upregulated mitophagy could have also contributed to the improved β‐cell function, as strengthened by regression analysis, which revealed a positive association of HOMA‐β indices with a key mitophagy player PINK1. This finding indicates that enhanced mitophagy in mononuclear cells provides the surrogate evidence of improvement in mitochondrial‐dependent regulation of β‐cell function, which may delay the progression of diabetes‐related complications.

Several studies have documented that chronic hyperglycaemia‐induced oxidative stress is associated with the development and progression of diabetes‐related complications.^37^, ^38^ Our results revealed that the metformin led to a significant decline in mtROS production and collapsed MMP in patients with NDT2DM. In consonance with our findings, Esteghamati et al (2015) demonstrated that metformin treatment for 12 weeks significantly decreased the oxidative stress levels in patients with newly diagnosed T2DM.^39^ Similarly, the use of voglibose in patients with NDT2DM was also associated with a significant reduction in the MOS parameters, whereas no significant alterations in MOS levels were noted in the placebo group. Collectively, our observations suggest that both metformin and voglibose therapy led to a reduction in glucotoxicity, consequently decreasing the MOS levels, although the magnitude of the decrease was greater in the metformin group.

Emerging lines of evidence suggest that the process of mitophagy is mediated via certain specific proteins that accelerate the removal of superfluous mitochondria in order to preserve healthy mitochondrial network. Therefore, we further investigated the effect of metformin on the expression profiling of mitophagy‐related markers in patients with NDT2DM, and our study is the first interventional trial to demonstrate the aforementioned findings. Interestingly, in the present study, NDT2DM patients treated with metformin displayed a significant upregulation in the mRNA and protein expression of mitophagy‐related genes including PINK1, PARKIN, MFN2, NIX, LC3‐II and LAMP2, as compared to the voglibose group, despite the fact that both metformin and voglibose monotherapies resulted in a similar degree of reduction in HbA_1C_ levels. These findings suggest that metformin significantly influenced the process of mitophagy de novo, and this propitious effect was observed to be independent of its efficacy on glycaemic control, in contrast to voglibose. In line with our findings, our previous in vitro study revealed that there was an increased formation of mitophagosomes as well as enhanced mitophagic flux in mononuclear cells following metformin exposure.^23^

Prior studies have documented that the pleiotropic effects of metformin are mediated via AMPK activation.^40^ A study by Musi *et al* (2002) suggested that 10‐week metformin treatment significantly augmented the AMPK activity in skeletal muscles of T2DM patients.^41^ Moreover, AMPK is also shown to be a positive regulator of autophagy. Our findings highlight that metformin caused a significant increase in p‐AMPKα (T172) levels, whereas voglibose did not display any effect on the same. Therefore, it is conceivable that metformin regulates mitophagy, which is mediated via the activation of the AMPK as also supported by our previous in vitro observations.^23^

Furthermore, TEM analysis demonstrated that following metformin treatment, there was reduced distortion in mitochondrial morphological characteristics including aspect ratio, form factor, circularity, interconnectivity and decreased degeneration of cristae, as compared to the voglibose and placebo group, depicting a healthy larger mitochondrial network with higher respiratory activity. These findings were further strengthened by the presence of a decreased percentage of damaged/dysfunctional mitochondria in these patients, representing a surrogate evidence of mitophagy flux. Thus, abovementioned observations suggest that metformin, beside an insulin sensitizer, also exerts its extended effect on the clearance of defunct mitochondria, thereby precluding the aggregation of these superfluous mitochondria, and subsequently mitigating the mitochondrial ROS production, which may otherwise severely  impair healthy mitochondria, leading to mitochondrial dysfunction.

**Figure 4:**
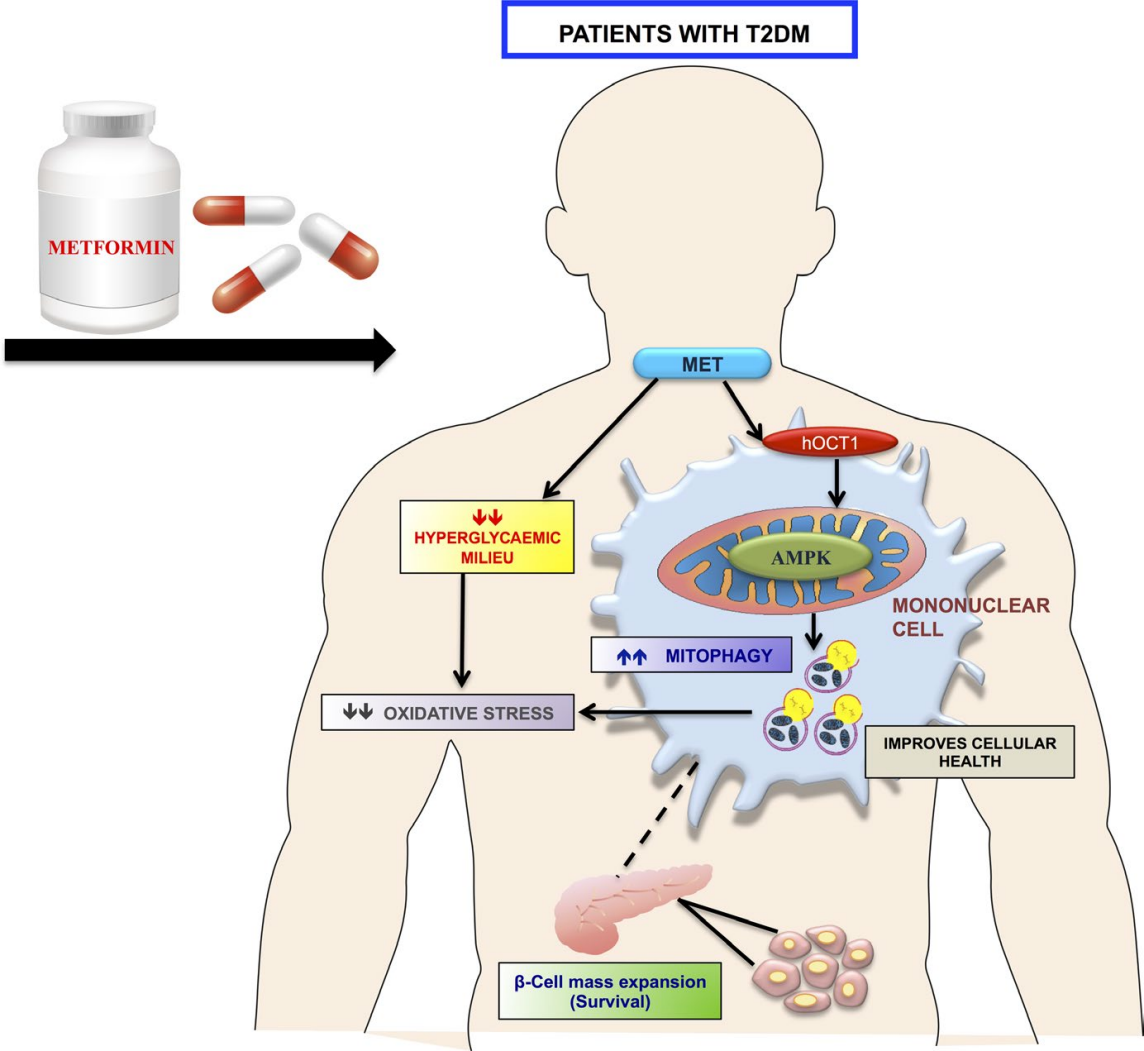
Depicting that metformin induces mitophagy via AMPK activation in mononuclear cells; a surrogate cellular model (indicated by dotted lines) for insulin‐sensitive sites, indicating improvement in cellular health including β‐cell function by containing oxidative stress‐mediated mitochondrial damage

Previous studies have reported that mitophagy negatively regulates inflammasome activation and metformin treatment decreases the expression of NLRP3, caspase‐1 and IL‐β in NDT2DM patients.^42^, ^43^ However, our data appeared inconsistent with prior studies and revealed that metformin therapy led to augmented NLRP3 protein expression in these patients, which may subsequently trigger caspase‐1‐mediated pyroptosis of chronically activated macrophages, and thereby impeding chronic inflammation (Figure S3). In support of this, it has been deduced that the extent of pyroptosis determines the magnitude of inflammation, and the controlled pyroptosis plays a crucial role in limiting the extent of inflammation and maintains a hemostasis state, as was observed in the early and chronic stage of apical periodontitis.^15^ Further, a study by Zha et al (2016) also reported that inflammasome activation was enhanced in metformin‐treated mice upon bacterial infection.^19^ Moreover, our observations revealed a strong and positive correlation of AMPK⍺(T172) with NLRP3 levels in the metformin group. Based on these findings, enhanced NLRP3 expression and increased mitophagy could be attributed to the metformin‐induced increased AMPK phosphorylation, as AMPK activation indirectly or directly regulates the phosphorylation of NLRP3 and mitophagic proteins. Therefore, it is a likelihood that metformin‐induced AMPK phosphorylation triggers mitophagy along with the NLRP3 activation, hence contributing towards the restoration of normal mitochondrial phenotype and alleviation of chronic inflammation by promoting pyroptosis in T2DM patients (Figure 4 and Figure S4).

Lastly, mitophagy and mitochondrial oxidative stress parameters were investigated in PBMCs, as in recent years, these cells have emerged as a surrogate model for insulin‐target sites for assessing the mitochondrial function in patients with T2DM.^21^, ^22^ In addition, these cells are easily accessible, express insulin receptors and readily respond to the circulating glucose and insulin concentrations.^20^ They are also known to exhibit alterations in several signalling transduction pathways, which are similar to those in β‐cells of patients with T2DM.^44^ Moreover, AMPK is ubiquitously expressed in mammalian cells and is one of the putative targets of metformin action, and further the presence of hOCT1 (human organic cation transporter 1) on PBMC’s plasma membrane, as also observed in our previous study, facilitates the uptake of metformin into these cells; therefore, it is apt to conclude that metformin is able to exert its effect on mitophagy via AMPK phosphorylation in mononuclear cells as well.^23^ In addition, augmented PINK1 expression with metformin therapy showed a significant association with HOMA‐β indices, indicating that hyperglycaemic milieu alters the gene expression profile in PBMCs, which may also be reflected in the insulin‐sensitive tissues, as strengthened by a couple of studies available in the literature.^45^, ^46^ Thus, the use of PBMCs as a cellular model may serve as an early diagnostic and prognostic tool in the management of diabetes.

The strengths of our study include a randomized controlled trial (RCT) design for assessing the effect of metformin on mitophagy, and the use of voglibose as an active comparator for abrogating the confounding effects of reduced glucotoxicity on mitophagy in patients with T2DM. Further, improvement in mitochondrial morphology and function following metformin therapy provides additional strength to our observations. Nevertheless, large cohort studies with a longer duration of follow‐up may reveal substantial beneficial effect of metformin on the restoration of normal mitochondrial architecture in patients with T2DM. However, the retrospective registration of our clinical trial remains a limitation of our study.

In conclusion, the present study highlights that both metformin and voglibose decreased glucotoxicity, mitochondrial oxidative stress indices, while only metformin therapy positively regulated mitophagy and inflammasome activation via the AMPK pathway. This cumulative effect exerted by metformin may prove favourable by culminating the damaged mitochondrial population, and by promoting pyroptosis to eliminate the ‘chronically activated’ macrophages, thereby reducing chronic inflammation and worsening of hyperglycaemia in T2DM patients. Furthermore, these findings provide novel mechanistic insights into the metformin action, besides its insulin‐sensitizing effect. In addition, mitophagy induction may serve as a novel therapeutic target for metformin action, which may also prove beneficial for treating mitochondrial‐related disorders.

## CONFLICT OF INTEREST

The authors declare that there is no duality of interest associated with this manuscript.

## AUTHOR CONTRIBUTIONS

SB designed and performed the experiments, acquired and analysed data, interpreted the results and drafted the manuscript. AB, PD and RW assigned the interventions to the patients. VD provided the laboratory facilities for conducting the research study. All the authors revised and approved the final version of the manuscript.

## Data Availability

The data sets supporting the conclusion of this work are included in the article and its supplementary files.
