# Supplementary material for: Metformin upregulates mitophagy in patients with T2DM: A randomized placebo‐controlled study
Source: J Cell Mol Med. 2020 Jan 23;24(5):2832–46. doi: 10.1111/jcmm.14834 (PMC7077543; doi:10.1111/jcmm.14834)
Supplement: Supplementary file 1 [file JCMM-24-2832-s001.doc]

**Fig. S1** Consort Patient Flow diagram for prospective, single-blind, randomized and placebo- controlled study (LSM=Lifestyle modifications).

**Fig. S2** Scatter plot showing mRNA expression of mitophagy-related genes (a) *MFN2 (b)PINK1 (c) PARKIN (d) NIX (e) LC3-II and (f) LAMP2*, assessed at baseline and after 3 months of respective treatments in the NDT2DM subjects. Horizontal lines represent median and interquartile range, (n= 13-15 each). P-values are for Wilcoxon Signed-rank test (baseline *vs.* post) ,*p<0.05; **p<0.01.

he above said award for her on 6th s, d relevant documentsch has Biomedical Sciences.sali for the above said award for her**Fig. S3** Representative western blot of caspase-1 in patients with NDT2DM, at baseline and at 3 months of respective therapies, (n=1 each).

he above said award for her on 6th s, d relevant documentsch has Biomedical Sciences.s

**Fig. S4** Proposed model illustrating meformin-mediated activation of NLRP3 inflammasome via AMPK phosphorylation, which may prove beneficial in patients with T2DM by promoting pyroptosis of “chronically-activated” macrophages, subsequently limiting chronic inflammation following metformin therapy.
