# Supplementary material for: Metformin upregulates mitophagy in patients with T2DM: A randomized placebo‐controlled study
Source: J Cell Mol Med. 2020 Jan 23;24(5):2832–46. doi: 10.1111/jcmm.14834 (PMC7077543; doi:10.1111/jcmm.14834)
Supplement: Supplementary file 2 [file JCMM-24-2832-s002.doc]

**Table S1: Comparative efficacy of Metformin vs. voglibose and placebo on mitochondrial oxidative stress indices**

|  | Baseline Model (n=42) | | | | | Controlling for Age | | Controlling for Age, BMI | | Controlling for Age, BMI, HbA_1c_ | | Controlling for Age, BMI, HbA_1c_, HOMA-IR | | Controlling for Age, BMI, HbA_1c_, HOMA-IR, HOMA-β | | | | |
| --- | --- | --- | --- | --- | --- | --- | --- | --- | --- | --- | --- | --- | --- | --- | --- | --- | --- | --- |
|  | P-Value | Effect  size (%)^a^ | Adjusted 3-months mean^b^  (95% CI) | | | P-Value | Effect  size (%)^a^ | P-Value | Effect  size (%)^a^ | P-Value | Effect  size (%)^a^ | P-Value | Effect  size (%)^a^ | P-Value | Effect  size (%)^a^ | Adjusted 3-months mean^b^  (95% CI) | | |
|  |  |  |  |  |  |  |  |  |  |  |  |  |  |  |  | Met | Vogli | Placebo |
|  |  |  | Met | Vogli | Placebo |  |  |  |  |  |  |  |  |  |  |  |  |  |
| mtROS | 0.171 | 8.9 | 151.2  (90.5, 212.0) | 148.9  (93.0, 204.8) | 221.8  (159.6, 283.9) | 0.103 | 11.6 | 0.100 | 12.0 | 0.115 | 11.6 | 0.143 | 11.8 | 0.390 | 6.7 | 151.6  (84.4, 218.8) | 167.8  (105.8,229.8) | 220.4  (150.8, 290.1) |
| MMP | 0.046 | 15 | 5.9  (3.7, 8.1) | 6.3  (4.1, 8.5) | 9.7  (7.3, 12.0) | 0.032 | 17.0 | 0.013 | 21.3 | 0.013 | 22.1 | 0.054 | 17.2 | 0.217 | 10.7 | 5.8  (3.3, 8.3) | 7.1  (4.7, 9.5) | 9.1  (6.5, 11.7) |

1. Variance explained by the use of metformin; calculated from partial eta squared. Squared values of 1%, 6%, and 13.8% indicate small, medium, large effect sizes respectively.
2. Adjusted 3-month mean for outcome variables calculated controlling for effects of confounder(s).

**Table S2: List of primary antibodies**

| **Antibody** | **Source** | **Dilutions** | **Manufacturer** | **Catalog No.** |
| --- | --- | --- | --- | --- |
| **p-AMPKα (T172)** | Rabbit polyclonal | 1:1000 | Cell Signaling Technology | 2535 |
| **PINK1** | Rabbit polyclonal | 1:1500 | Sigma-Aldrich | p0076 |
| **PARKIN** | Rabbit polyclonal | 1:2000 | Abcam | ab15954 |
| **NIX** | Rabbit polyclonal | 1:2000 | Abcam | ab8399 |
| **MFN2** | Mouse polyclonal | 1:1000 | Abcam | ab56889 |
| **LC3-II** | Rabbit polyclonal | 1:1000 | Sigma-Aldrich | L7543 |
| **LAMP2** | Rabbit polyclonal | 1:700 | Abcam | ab37024 |
| **NLRP3** | Rat polyclonal | 1:1000 | R & D systems | MAB7578 |
| **β-ACTIN** | Goat polyclonal | 1:1000 | Abcam | ab8229 |

**Table S3: Human-specific primer sequences for qRT-PCR**

| **Primer name** | **Sequences** |
| --- | --- |
| **PINK1** | F-5’-GGGGAGTATGGAGCAGTCAC-3’  R-5’-CATCAGGGTAGTCGACCAGG-3’ |
| **PARKIN** | F-5’-TACGTGCACAGACGTCAGGAG-3’  R-5’-GACAGCCAGCCACACAAGGC-3’ |
| **MFN2** | F-5’-TGATGGGCTACAATGACCAG-3’  R-5’-AGCTTCTCGCTGGCATGC-3’ |
| **NIX** | F-5’-AAAATGAGCAGTCTCTGCCCC-3’  R-5’-TGCTGCTGTTCATGGGTAGCT-3 |
| **LC3-II** | F-5’-CCACACCCAAAGTCCTCACT-3’  R-5’-CACTGCTGCTTTCCGTAACA-3’ |
| **LAMP2** | F-5’-CGTTCTGGTCTGCCTAGTCC-3’  R-5’**-**CAGTGCCATGGTCTGAAATG-3’ |
| **β-ACTIN** | F-5’- GGCACCCAGCACAATGAAG- 3’  R-5’ -AGCTTCTCGCTGGCATGC-3’ |
